# Social health indicators promoting quality of life for the older people in the Kingdom of Saudi Arabia

**DOI:** 10.3389/fpubh.2026.1741815

**Published:** 2026-03-11

**Authors:** Hanaa Faize A. Moubarak, Basheer Ali Allouash, Abeer Niazi Wajeed Fathallah, Asyraf Afthanorhan

**Affiliations:** 1 Department of Social Sciences, College of Arts, University of Ha’il, Ha'il, Saudi Arabia; 2 Humanities Research Center, University of Ha’il, Ha’il, Saudi Arabia; 3 Faculty of Business and Management, Universiti Sultan Zainal Abidin (UniSZA), Kuala Terengganu, Malaysia

**Keywords:** older people, quality of life, social adjustment, social health, social support

## Abstract

**Introduction:**

This study examines the contribution of social health to enhancing the quality of life (QOL) among older people in the Kingdom of Saudi Arabia. Given that QOL is a critical health indicator in aging populations, understanding the role of social health is essential for supporting well‑being in later life.

**Methods:**

A descriptive research design was employed using the Social Health Scale for Older People. The study sample consisted of 660 Saudi participants aged 65 years and above, representing 0.1% of the national older population.

**Results:**

It revealed a high overall mean for social health indicators, particularly in the domains of social support (M = 2.6341) and social adjustment (M = 2.4066). Social support included emotional acceptance, comfort, encouragement, and daily care. Social adjustment encompassed employment, household involvement, interpersonal communication, psychological well‑being, and self‑health awareness. The results indicate that older people in Saudi Arabia benefit from strong social health outcomes influenced by cultural values, family‑centered care, and positive youth attitudes.

**Discussion:**

Despite the current strengths in social health, anticipated demographic changes are expected to significantly increase the number of older people in the coming years. This highlights the need for proactive policy interventions. The study recommends strengthening family‑based support through community initiatives, expanding access to institutional care, and promoting intergenerational awareness to ensure sustainable well‑being and dignity for older people in Saudi society.

## Introduction

The global population of older adults is rapidly increasing, driven by medical advancements that have significantly extended human longevity. According to the United Nations, declining birth and death rates have led to a marked rise in the proportion of older people across all cultures and societies—a trend expected to persist well into the next century. Between 2000 and 2050, the number of people aged 60 and above is projected to triple, reaching at least two billion. Notably, the segment aged 80 and above is growing at the fastest rate, while the number of centenarians is expected to rise fourteen-fold, from 265,000 in 2005 to 3.7 million by 2050. This demographic shift is particularly pronounced in developing countries, where aging populations pose additional challenges to economic development and social support systems (1–3, 51).

Caring for the older people has thus emerged as a pressing global concern—one that is multidimensional, humanitarian, and social in nature. Societies, regardless of their scientific, cultural, or economic advancement, face increasing pressure to provide adequate care for their aging populations. Older people care is a cornerstone of state-level social welfare policy, requiring the integration of older adults into society in ways that affirm their dignity, purpose, and capabilities (52).

In Saudi Arabia, although the older people represent only 3.5% of the total population—compared to the global average of 10%—the government has proactively invested in initiatives aimed at enhancing their quality of life. These efforts include the establishment of 12 social care homes across the Kingdom, offering comprehensive health, psychological and social services. Older people are encouraged to engage in voluntary activities, such as intergenerational storytelling and community service. Financial and material support is also provided through the Social Security Agency, while the private sector has contributed by founding 13 civil societies dedicated to older people care (53, 54).

The Kingdom’s commitment to older people’s welfare is enshrined in Article 27 of the Basic Law of Governance, which guarantees citizens’ rights in cases of emergency, illness, disability, and old age. It also mandates the provision of public health services to all citizens. Further reinforcing this commitment, the “Rights and Care of the Older people” law—issued under Royal Decree No. (M/47) dated 3/6/1443 AH—aims to safeguard the dignity, privacy, and independence of older adults while ensuring access to essential services (55).

The World Health Organization (WHO) identifies quality of life (QoL) as a key metric for evaluating the aging process, particularly among older adults (4). QoL assessments are widely used in gerontological research to measure functional status, especially in individuals with chronic conditions (5). Given its multidimensional nature, QoL encompasses physical, emotional, cognitive, social, and economic domains, offering a holistic view of well-being in later life (6).

Conceptualizing Quality of life (QoL) in older age is essential, as it reflects individuals’ perceptions of their living standards across various domains (7). Scholars distinguish between social indicators, objective conditions or experiences—and psychological indicators, which capture subjective feelings about those conditions (8). Despite extensive research, there remains no universally accepted definition of QoL in old age. Comprehensive assessments should include aspects such as physical and sexual health, emotional and behavioral functioning, cognitive and intellectual engagement, social support, life satisfaction, perceived health, recreational capacity, vitality, and financial stability (56).

Among these dimensions, social health plays a pivotal role. Closely linked to emotional and mental well-being, social health directly influences physical outcomes; poor social health has been associated with hypertension, cardiovascular disease, and psychological distress (9). The quality of interpersonal relationships—defined by depth and meaningful connection rather than quantity—is a critical determinant of social health (10). As Maxwell (11) suggests, the universal aspiration for a healthy society is rooted in the pursuit of strong social health and a high quality of life.

Considering these considerations, the present study seeks to examine the social health indicators that contribute to enhancing the quality of life among older people in the Kingdom of Saudi Arabia.

## Literature review

### Quality of life

Asiri et al. (12) examined the relationship between perceived social support, religious faith, and quality of life (QoL) among hemodialysis patients in Aseer, Saudi Arabia. Their findings revealed a significant positive correlation between perceived social support, religious faith, and life satisfaction (*p* < 0.05). The study concluded that both factors play a critical role in enhancing health-related quality of life (HRQoL), underscoring the importance of integrating psychosocial and spiritual assessments into patient care. Al-Husseini (13) explored the conceptual dimensions of QoL and its determinants, comparing them with the objectives of the Kingdom’s Vision 2030 Quality-of-Life Program. The study emphasized the alignment between national policy initiatives and patient-centered care within the healthcare system.

Focusing on older women in Riyadh, Alsheikh (14) investigated the impact of family support on QoL. The results indicated a direct positive relationship, suggesting that increased familial engagement—particularly considering ongoing societal, economic, and technological changes—can significantly enhance QoL among older women. Al-Shammari (15) sought to define the components of successful aging and QoL among the older people. Although the study found that many participants did not experience successful aging due to economic and health-related challenges, their QoL remained relatively high. This was attributed to personal growth, psychological resilience, and the cultivation of meaningful interpersonal relationships, which collectively contributed to a sense of purpose and well-being.

Haddah (16) addressed the paradoxical interplay between aging, illness, and happiness. The study highlighted efforts to combat age-based discrimination and promote inclusive services for the older people. It also advocated for active aging through physical activity, enabling older adults to maintain societal engagement and benefit from healthcare networks. Mourad et al. (17) assessed QoL among residents of an older people care facility, reporting that 70% had low QoL and 85% lacked awareness of the aging process. The study identified a negative correlation between QoL and both gender and income source, while education level and frequency of visitors were positively associated with better QoL outcomes.

Amir et al. (18) examined the effects of daily religious practices on QoL and cognitive function among the older people. Their findings demonstrated that regular religious engagement significantly improved both domains, reinforcing the role of spirituality in promoting healthy and successful aging. El-slamoni and Abdel Karim (19) evaluated the impact of reminiscence therapy on depression, loneliness, and QoL in older adults. The intervention was found to significantly reduce depressive symptoms and feelings of isolation, thereby enhancing overall QoL. Gunawan et al. (20) explored predictors of QoL among older people individuals in nursing homes. Key variables included age, gender, education, marital status, ethnicity, chronic illness, and depression. Among these, age, depression status, and educational attainment emerged as the most significant predictors of QoL.

### Social health

Alidoust and Bosman (21) conducted a critical review of literature on how urban neighborhood environments influence the social lives of older adults. Their proposed framework emphasized the importance of safe, accessible, walkable, and green neighborhoods in fostering social engagement and enhancing social health among the older people. Khattab (22) found that older women reported higher HRQoL than men, particularly in areas such as general health, daily activity, vitality, and social functioning. Women also benefited from stronger social networks. The study concluded that various forms of social support—emotional, instrumental, and network-based—significantly influence awareness and perception of HRQoL in older populations, regardless of gender. Dhingra (23) highlighted growing concerns about social health, noting that challenges such as dishonesty, unrealistic expectations, low self-esteem, and jealousy undermine interpersonal relationships. The study identified social competence and social support as essential components of individual social health.

Collectively, the reviewed studies underscore the multidimensional nature of quality of life (QoL) among older people, shaped by psychosocial, spiritual, and structural factors. Religious engagement and family support consistently emerge as key enhancers of QoL, while variability in outcomes across institutional settings (e.g., nursing homes vs. family households) highlights the need for context-sensitive interventions. The emphasis on personal growth and social connectedness aligns with Bourdieu’s notion of social capital, suggesting that QoL is not merely a health metric but a reflection of embedded social resources. These findings reveal a complex interplay between individual agency, cultural norms, and structural conditions in shaping both QoL and social health. Yet, gaps persist in understanding how these dynamics manifest within specific regional contexts—such as Ha’il—and how they intersect with broader national initiatives like Vision 2030. This study aims to address these gaps by examining the sociocultural dimensions of aging in Saudi Arabia, with particular attention to the role of symbolic and social capital in shaping older people experiences.

### Questions

Main Question: What are social health indicators promoting quality of life for the older people in the Kingdom of Saudi Arabia?

Sub-Questions:

1) What are social support indicators promoting quality of life for the older people in the Kingdom of Saudi Arabia?
2) What are social adjustment indicators promoting quality of life for the older people in the Kingdom of Saudi Arabia?

## Definitions

Social health refers to an individual’s capacity to establish and maintain healthy, rewarding interpersonal relationships (10). It encompasses the ability to adapt to social changes, set appropriate boundaries within relationships, and balance personal and social commitments. The World Health Organization (WHO), in its 1948 constitution, defined health as “a state of complete physical, mental, and social well-being, and not merely the absence of disease or infirmity,” thereby recognizing the integral role of social well-being in overall health.

The context in which individuals live, including their social, economic, and physical environments, has a profound impact on their health and quality of life. According to WHO, these environmental factors, along with personal characteristics and behaviors, are key determinants of health. Thus, social health involves not only the ability to form satisfying relationships but also the capacity to navigate diverse social situations and respond appropriately across settings.

In this regard, fostering healthy interpersonal dynamics among spouses, neighbors, colleagues, and peers is essential. Such relationships should be grounded in effective communication, empathy, and mutual accountability (23). Within the context of aging, the current study defines social health among the older people as “presence of perceived social support and an ability for social adjustment for the older people.”

Quality of life (QoL) is defined by the World Health Organization (WHO) as “an individual’s perception of their position in life in the context of the culture and value systems in which they live and in relation to their goals, expectations, standards, and concerns” (24). This definition emphasizes the subjective and culturally embedded nature of QoL, extending beyond physical health to encompass psychological, social, and environmental dimensions. In the context of aging, QoL among the older people is commonly assessed through indicators such as levels of depression, perceived health status, financial satisfaction, quality of relationships with spouse and children, sexual activity, and engagement in leisure pursuits. These factors are shaped by cumulative life experiences, including decisions and events across the life course, as well as the influence of one’s living environment and lifestyle (25).

The term older people are conventionally used to describe individuals aged 65 years and older. Within this classification, those aged 65 to 74 are referred to as the “early older people,” while individuals aged 75 and above are considered the “late older people” (26).

Social support, often conceptualized more broadly as a form of *social capital*, encompasses multiple dimensions. The first is the structural aspect, which refers to the support received through an individual’s social network. This dimension is influenced by the size of the network and the degree of connectivity within it. For instance, individuals with a large number of social ties may engage in frequent and meaningful interactions—such as emotional exchanges, practical assistance, or shared activities—that contribute to their overall well-being. Strong relationships with family and friends are particularly vital for fostering successful social integration.

The second dimension is the functional or qualitative aspect, which pertains to an individual’s subjective perception of the support they receive and their sense of integration within the social network (27). This includes not only the availability of support but also its emotional significance and perceived reliability.

Song et al. (28) define social support as “an enduring pattern of continuous or intermittent ties that play a significant part in maintaining the psychological and physical integrity of the individual over time.” They identify three core functions of social support: (1) mobilizing psychological resources and managing emotional burdens, (2) sharing tasks and responsibilities, and (3) providing material, cognitive, and instrumental resources—such as money, tools, skills, and guidance—to help individuals navigate life challenges.

Social adjustment refers to the process by which individuals align their behaviors, values, and interactions with the accepted norms and expectations of their society. It involves the capacity to “get along with members of society as best as one can,” reflecting an individual’s ability to function harmoniously within various social contexts (57). Successful adjustment to one’s environment is closely linked to enhanced self-worth, particularly when individuals invest their energy in navigating social expectations. As Alam (29) notes, social adjustment encompasses the development of positive and adaptive relationships within one’s immediate social sphere, including the home, peer groups, cultural settings, and the broader community, based on prevailing social norms and requirements.

## Theoretical framework

Contemporary research on quality of life (QoL) distinguishes between objective and subjective indicators. Objective QoL encompasses externally measurable conditions such as material wealth, social status, and physical health—elements often shaped by societal and cultural expectations (58). These indicators can be quantified and compared across populations. In contrast, subjective QoL reflects an individual’s internal evaluation of life circumstances, including emotional satisfaction, perceived well-being, and personal fulfillment, which are accessible only through self-reported experiences.

The spillover theory offers a dynamic understanding of QoL by positing that satisfaction in one life domain influences satisfaction in others. Sirgy et al. (59) propose a hierarchical model of life domains—such as family, work, health, and leisure—with general QoL at the apex. Positive experiences in lower domains can “spill over” to enhance overall life satisfaction, suggesting an interdependent structure of well-being.

From a cognitive perspective, QoL is shaped primarily by individual perception. This view asserts that (1) an individual’s subjective interpretation of life circumstances determines their sense of QoL, and (2) due to cognitive variability, subjective factors often exert a stronger influence than objective ones. Schalock et al. (30) conceptualize QoL as a multidimensional construct comprising eight domains: emotional well-being, interpersonal relationships, material well-being, personal development, physical well-being, self-determination, social inclusion, and rights.

To deepen this framework, Bourdieu’s theory of capital provides a sociological lens through which QoL can be understood as a product of accumulated resources. Bourdieu identifies four forms of capital—economic, cultural, social, and symbolic—that shape individuals’ life chances and social positioning. In the context of aging, social capital (e.g., family ties, community networks) and symbolic capital (e.g., respect, status) play a critical role in determining how older people experience QoL. The ability to mobilize these forms of capital influences access to support, autonomy, and recognition within society.

Together, these theoretical perspectives provide a robust foundation for examining QoL among older people in Saudi Arabia. They illuminate how subjective experiences, structural conditions, and symbolic practices interact to shape well-being, and offer a nuanced framework for analyzing the sociocultural dimensions of aging in relation to Vision 2030 and regional dynamics such as those in Ha’il.

## Methodology

### Study design and population

This study adopts a descriptive research design aimed at monitoring key indicators of social health that contribute to the quality of life (QoL) among older people in the Kingdom of Saudi Arabia. The research is based on a social survey approach, targeting a representative sample of Saudi families.

According to the 2022 census conducted by the General Authority for Statistics, the Saudi population aged 65 years and above totals 659,871 individuals. A stratified sample comprising 0.1% of this population was selected, resulting in a final size sample of 660 older people participants. The sample was proportionally distributed across three age categories in Table 1:

**Table 1:** Distribution of the study sample.

Age category	Population	Sample size
65–69 years	257,136	258
70–74 years	155,576	156
75 + years	247,159	247
Total	659,871	660

Data was collected from the target population through the following procedures:

1) Social work students were engaged to reach older people in hospitals, health centers, social care homes, and charitable institutions and associations where they conduct their field training. In addition, older people were approached within their families, among friends, and neighbors.
2) Approximately 1,050 older people were contacted, of whom 660 responded. Most respondents were women (74.5%), while about 390 older people—mostly men—either declined participation or delayed their response beyond the data collection period.
3) This situation imposed certain constraints, rather than bias, on the study sample, specifically related to:

(i) Gender: 74.5% female and 25.5% male. Women demonstrated greater enthusiasm and cooperation compared to men, who showed less interest and engagement. This distribution contrasts with the official demographic statistics of older people Saudis reported by the General Authority for Statistics (57% male, 43% female).
(ii) Educational status: Educated participants showed higher willingness and cooperation, with 45% holding a bachelor’s degree. Nevertheless, the study sample also included illiterate individuals and those with below average or average levels of education.

### Research instrument

Data was collected using the Social Health Scale for the Older people, originally developed by Bao et al. (31). The full scale comprises three primary dimensions—social support, social adjustment, and perceived environmental resources—organized into nine sub-dimensions and a total of 40 items. For the purposes of this study, the third dimension (*perceived environmental resources*)—including its three sub-dimensions and ten items—was excluded due to its limited applicability within the Saudi sociocultural context. The adapted instrument therefore focused on two core dimensions:

- *Social Support*: Emotional, informational, and instrumental support (15 items).
- *Social Adjustment*: Social participation, social relationships, and ego system (15 items).

It should be noted that the instrument was culturally adapted to fit the Saudi context in accordance with the recommended scientific procedures. The scale was first translated into Arabic and then back translated into English by bilingual experts to ensure semantic equivalence. Furthermore, the items of the scale were reviewed by a panel of specialists in public health, social health, social work, and psychology to confirm their cultural appropriateness and clarity. A pilot test was also conducted on a small sample of older adults to verify ease of comprehension and the suitability of the items.

Responses were measured using a 3-point Likert scale, where: 3 = Good, 2 = Acceptable, and 1 = Poor. The instrument’s construct validity was assessed using correlation coefficients, yielding a statistically significant value of 0.754 (*p* < 0.01). Internal consistency reliability was confirmed through Cronbach’s Alpha, which produced a coefficient of 0.892, indicating high reliability.

## Results and discussion

### Description of the research sample

Table 2 outlines the demographic and socioeconomic characteristics of the study sample. Most participants were female (74.5%), while males constituted only 25.5%. Educational attainment was generally high: 45% held a bachelor’s degree, 20.5% had completed intermediate education, 15.2% had pre-intermediate education, and 0.9% held a master’s degree. The proportion of uneducated individuals did not exceed 18.5%. In terms of marital status, 60.3% of respondents were single, compared to 38% who were married. Regarding economic conditions, 75% reported having an average income level. Housing data revealed that 87.9% lived in rental accommodations, and employment figures showed that 88.8% were not engaged in formal work. Health status was predominantly positive, with 78.8% indicating they were in good health. Overall, the sample is characterized by a predominance of educated women with moderate economic means, good health, and a high rate of singlehood. The gender imbalance in participation may be attributed to a greater willingness among women to engage in the study compared to men.

**Table 2:** Characteristics of the research sample.

Variables (*N* = 660)		*N*	%
Gender	Male	168	25.5%
	Female	492	74.5%
Educational status	Uneducated	122	18.5%
	Pre-intermediate education	100	15.2%
	Intermediate education	135	20.5%
	Bachelor’s	297	45%
	Master’s	6	0.9%
Social status	Single	398	60.3%
	Married	262	39.7%
Economic status	Limited	103	15.6%
	Middle	495	75%
	Rich	62	9.4%
Residential status	Rented	580	87.9%
	Owned	80	12.1%
Employment status	Working	74	11.2%
	Not-Working	586	88.8%
Health status	Good	520	78.8%
	Suffering from chronic diseases	132	20%
	Degraded	8	1.2%

### Social support indicators promoting quality of life for the older people in the Kingdom of Saudi Arabia

The findings presented in Table 3 reveal a relatively high overall mean score for emotional support among the older people in Saudi Arabia (M = 2.6345), suggesting its perceived significance in enhancing quality of life. Among the eight measured indicators, *total acceptance* ranked highest (M = 2.78), followed by *comfort* (M = 2.73), *companionship* (M = 2.68), and *listening* (M = 2.66). These were closely followed by *emotional care* (M = 2.65), *support in major decisions* (M = 2.63), and *understanding* (M = 2.60), while *movement* received the lowest rating (M = 2.35).

**Table 3:** Indicators of emotional support.

Items (*N* = 660)	Quality			Descriptive statistics		
	Poor	Acceptable	Good	Mean	Std. deviation	Rank
1. Being listened to	18	190	452	2.66	0.529	4
2. Being totally accepted	8	128	524	2.78	0.442	1
3. Being understood	32	202	426	2.60	0.581	7
4. Being supported in major decisions	20	206	434	2.63	0.543	6
5. Being cared for emotionally	20	192	448	2.65	0.538	5
6. Being accompanied	28	152	480	2.68	0.549	3
7. Being comforted	10	156	494	2.73	0.476	2
8. Being moved	120	192	348	2.35	0.769	8
Average mean = 2.6345 (High)						

0.00–1.00 = Low; 1.01–2.00 = Moderate; 2.01–3.00 = High.

This ranking pattern suggests that emotional support dimensions rooted in interpersonal affirmation and empathetic engagement such as acceptance, comfort, and active listening—are prioritized over functional or physical assistance. Such preferences may reflect the psychosocial vulnerabilities associated with aging, including increased susceptibility to depression, anxiety, and feelings of isolation. These vulnerabilities are often exacerbated by declining mobility, chronic illness, cognitive impairment, bereavement, reduced income, and post-retirement identity loss. The elevated valuation of emotional support by the study sample aligns with Devkota et al. (32), who emphasize its critical role in enhancing older adults’ subjective health and overall psychological, social, and emotional well-being. It is plausible that these emotional dimensions are universally beneficial across age groups, yet their salience intensifies in later life due to compound existential and health-related stressors. From a sociological perspective, this underscores the importance of culturally attuned support systems that prioritize emotional connectivity as a cornerstone of elder care in Saudi society.

Bourdieu’s theory of capital offers a multidimensional lens through which emotional support for the older people can be understood. Among his forms of capital, economic, cultural, social, and symbolic—social capital is particularly relevant to this study. It refers to the resources embedded in social networks and relationships, including trust, mutual recognition, and obligations. In the context of aging, emotional support functions as a vital form of social capital, sustaining the older people’s sense of belonging, identity, and access to care. The indicators ranked highest in Table 3, *total acceptance*, *comfort*, *companionship*, and *listening* are not merely interpersonal gestures; they represent relational assets that reinforce the older people’s embeddedness within their social field. These forms of support reflect dense social ties and reciprocal recognition, which are central to Bourdieu’s conception of social capital. As physical and economic capital often decline with age, emotional support becomes a convertible resource, enabling older adults to maintain symbolic status and influence within family and community structures.

Moreover, the high valuation of emotional support by the Saudi sample may reflect a culturally specific habitus, a system of dispositions shaped by historical and social conditions. In Saudi society, where familial cohesion, intergenerational respect, and emotional proximity are deeply valued, the habitus predisposes individuals to prioritize emotional forms of care. This reinforces the idea that emotional support is not only a personal need but also a socially structured expectation, embedded in the moral economy of care. From a Bourdieuian perspective, emotional support thus operates as a non-material capital that preserves the older people’s position within the social hierarchy. It mediates access to symbolic recognition, decision-making inclusion, and psychological well-being. In this way, emotional support is both a product of social structure and a mechanism for its reproduction, making it a critical variable in understanding aging and quality of life in the Saudi context.

The results in Table 4 indicate a relatively high average mean for older people informational support (M = 2.6606), suggesting a consistent need for cognitive and guidance-based assistance among older adults. Among the indicators, “reminded to improve” ranked highest (M = 2.72), followed by “given useful suggestions” (M = 2.68), and “given useful information” (M = 2.58). While all three reflect dimensions of informational support, the prominence of “reminded to improve” warrants deeper interpretation. Unlike conventional informational support, which centers on the transmission of factual or procedural knowledge, this indicator implies a hybrid form of support—one that blends informational cues with emotional reinforcement and symbolic recognition.

**Table 4:** Indicators of informational support.

Items (*N* = 660)	Quality			Descriptive statistics		
	Poor	Acceptable	Good	Mean	Std. deviation	Rank
1. Being given useful information	8	262	390	2.58	0.518	3
2. Being reminded to improve	14	156	490	2.72	0.494	1
3. Being given useful suggestions	8	194	458	2.68	0.491	2
Average mean = 2.6606 (High)						

0.00–1.00 = Low; 1.01–2.00 = Moderate; 2.01–3.00 = High.

From a Bourdieusian perspective, this pattern may reflect the interplay between symbolic capital and habitus in later life. The act of reminding someone to improve is not merely informational—it signals care, recognition, and a reaffirmation of the older people’s social worth within their relational field. Such gestures may reinforce their position in the social space, especially in cultures where aging is associated with declining visibility. Moreover, the reception and valuation of such support may be shaped by the older people’s internalized dispositions (habitus), which influence how they interpret and respond to different forms of assistance. Alternatively, through Veblen’s lens, one could argue that informational support, particularly when framed as improvement, may function as a subtle form of conspicuous care. In contexts where caregiving itself is socially visible, the act of offering improvement-oriented reminders may serve as a status signal for caregivers, especially in familial or institutional settings. This interpretation aligns with Veblen’s notion that social actions, even altruistic ones, can carry performative and comparative dimensions. Cui et al. (33) underscores the importance of informational support in transitional care for older adults with chronic conditions, reinforcing the idea that such support is not only functional but also socially embedded. Thus, the data in Table 4 reveal more than just a hierarchy of informational needs—they reflect the symbolic and emotional textures of aging, care, and recognition within a sociocultural framework.

The findings presented in Table 5 indicate a notably high average score for instrumental support among the older people (M = 2.6072). Instrumental support encompasses provisions that address fundamental needs—such as financial assistance, material resources, help with household tasks, and caregiving during illness, which collectively contribute to emotional and social stability in later life. Within the sample, the highest-rated indicator was “cared for in daily life” (M = 2.72), followed by “helped with daily chores during illness” (M = 2.69), “given financial aid” (M = 2.53), and “given material aid” (M = 2.49). These results suggest that older people in Saudi Arabia receive substantial practical support, particularly in domains that ensure continuity of daily functioning and wellbeing.

**Table 5:** Indicators of instrumental support.

Items (*N* = 660)	Quality			Descriptive statistics		
	Poor	Acceptable	Good	Mean	Std. deviation	Rank
1. Being helped with daily chores during illness	36	132	492	2.69	0.568	2
2. Being given financial aid	72	165	423	2.53	0.684	3
3. Being given material aid	89	158	413	2.49	0.721	4
4. Being cared for in daily life	28	132	500	2.72	0.538	1
Average mean = 2.6072 (High)						

0.00–1.00 = Low; 1.01–2.00 = Moderate; 2.01–3.00 = High.

This elevated level of instrumental support may be attributed to the predominance of middle-class respondents, whose educational attainment and moderate-income levels afford them access to supportive resources. It is also important to contextualize these findings within the broader socioeconomic landscape of Saudi Arabia, where high per capita income and state-led initiatives aimed at enhancing social protection—especially for the older people—play a significant role. The prominence of the “cared for in daily life” indicator is further illuminated by labor statistics: in 2023, the number of domestic workers in Saudi Arabia reached approximately 3.64 million, with 1.75 million employed specifically in household cleaning roles (60). The widespread employment of domestic help across Saudi households reflects both cultural norms and structural conditions that facilitate high levels of instrumental support. Moreover, the cultural and religious emphasis on elder care in Islamic tradition reinforces the societal value placed on supporting older family members.

The high levels of instrumental support observed among older people Saudis can be interpreted through Bourdieu’s concept of economic capital, which refers to material resources that enable access to goods and services. In this context, the ability of families to provide financial aid, material support, and caregiving—often through domestic workers—reflects the mobilization of economic capital to secure stability and comfort for older family members. The prevalence of domestic labor in Saudi households, supported by middle-class income levels, illustrates how economic capital is converted into caregiving practices that reinforce familial roles and social cohesion. Beyond material resources, cultural capital plays a pivotal role in shaping caregiving norms. Bourdieu distinguishes between embodied, objectified, and institutionalized forms of cultural capital, all of which are relevant here. Embodied cultural capital is evident in the internalized dispositions and values that prioritize elder care, deeply rooted in Islamic teachings and Saudi social traditions. These values guide behavior and legitimize caregiving as a moral and social imperative. Objectified cultural capital may be reflected in the structured routines and household arrangements that facilitate elder support, while institutionalized cultural capital is reinforced through state-led initiatives aimed at social protection.

Social capital, defined by Bourdieu as the resources embedded in social networks and group membership, further illuminates the mechanisms of support. In Saudi society, dense kinship ties and communal structures foster reciprocal caregiving relationships. The older people benefit from being embedded in networks that valorize intergenerational responsibility, where support is not only expected but symbolically rewarded. This relational embeddedness enhances the availability and consistency of instrumental support, especially in times of illness or vulnerability.

The family, as a field in Bourdieu’s framework, serves as a structured social space where agents (family members) occupy positions and engage in practices shaped by their capital endowments and habitus. Within this field, caregiving emerges as a form of symbolic labor that reinforces familial hierarchies and social roles. The observed patterns of support reflect a habitus attuned to caregiving—an internalized system of dispositions cultivated through religious, cultural, and social experiences. This habitus predisposes individuals to act in ways that align with societal expectations of elder care, often without conscious deliberation. Finally, the provision of instrumental support can be understood as a form of symbolic capital, wherein caregiving practices confer moral prestige and social recognition. In a society where elder care is culturally valorized, families that actively support their older people distinguish themselves as ethically and socially aligned with dominant norms. This symbolic dimension reinforces the legitimacy of caregiving and elevates its status within the social hierarchy, contributing to the reproduction of cultural values across generations.

**Figure 1:**
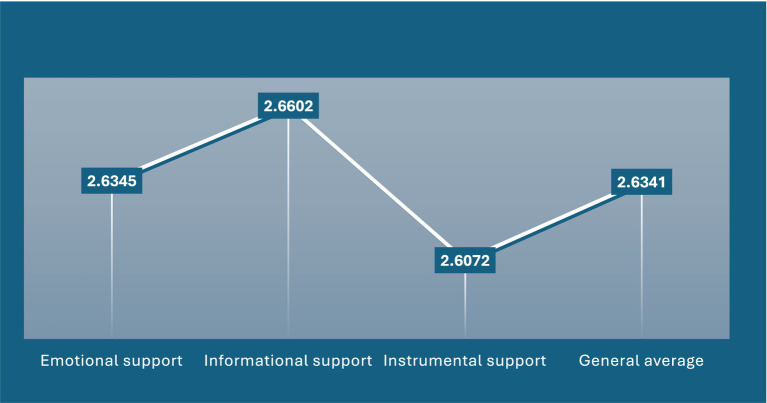
Social support indicators.

Figure 1 clearly illustrates that the overall dimension of social support is notably high, encompassing its three sub-dimensions—emotional, informational, and instrumental—along with their respective indicators. Collectively, these forms of support contribute significantly to enhancing the quality of life for older people in the Kingdom of Saudi Arabia. Social support plays a vital role in sustaining multiple aspects of an older people person’s life, thereby directly influencing their overall well-being. Gupta et al. (34) found that increased social support, particularly when provided by family members, is positively associated with improved psychological well-being among older adults. As individuals age, their vulnerability to physical and emotional challenges increases, making the reinforcement of social support essential to achieving optimal levels of happiness and life satisfaction.

Ahmed and Mohamed (35) emphasized that social support is a critical factor in preventing physical, psychological, and social difficulties. Their study concluded that strengthening social support and fostering feelings of happiness can enhance moral engagement and emotional resilience among older people. Similarly, Hosseini et al. (36) identified social support as a key lifestyle variable that contributes to reducing depression and stress in older populations. Furthermore, Liao et al. (37) demonstrated that providing instrumental support not only benefits the recipient but also positively impacts the provider, potentially extending life expectancy among older people.

### Social adjustment indicators promoting quality of life for the older people in the Kingdom of Saudi Arabia

The findings presented in Table 6 indicate a relatively high overall mean score for social participation among older people (M = 2.1114), reflecting an upward trend in the general indicators of social engagement. “Working for pay” emerged as the highest-ranked activity (M = 2.29), attributable to the 11.2% of participants who remained employed. “Doing housework” followed closely (M = 2.23), likely influenced by the predominance of female respondents (74.5%) in the sample. “Doing volunteer work” (M = 1.98) and “participating in collective recreational activities” (M = 1.95) ranked third and fourth, respectively. Notably, these two indicators were the only ones among the thirty assessed social health dimensions to fall within the medium range, underscoring the importance of integrating volunteering and recreational engagement into older people care strategies within Saudi society.

**Table 6:** Indicators of social participation.

Items (*N* = 660)	Quality			Descriptive statistics		
	Poor	Acceptable	Good	Mean	Std. deviation	Rank
1. Working for pay	132	207	321	2.29	0.778	1
2. Doing housework	144	221	295	2.23	0.783	2
3. Doing volunteer work	248	179	233	1.98	0.854	3
4. Participating in collective recreational activity	266	159	235	1.95	0.871	4
Average mean = 2.1114 (High)						

0.00–1.00 = Low; 1.01–2.00 = Moderate; 2.01–3.00 = High.

Social participation is widely recognized as a cornerstone of healthy aging. Manijeh and Farahnaz (38) emphasized that older adults place significant value on community involvement, particularly activities that foster interpersonal interaction and resource exchange, which contribute to personal fulfillment. Empirical evidence supports the positive association between social participation and various dimensions of well-being—individual, environmental, and social (39, 40). Baeriswyl and Oris (41) further demonstrated that engaging in multiple forms of social participation yields greater life satisfaction than singular involvement. Interactions within associations and visits from family and acquaintances were perceived as more meaningful than institutional or private forms of participation. For vulnerable populations, traditional modes of engagement, especially familial and religious, remain vital. In the Saudi context, Hawasheen and Hijazi (42) highlighted the diversity of participatory forms among older people men and women and reported a strong positive correlation between social participation and life satisfaction.

From a Bourdieusian perspective, these findings can be interpreted through the lens of social capital and habitus. Social participation, whether through paid work, domestic labor, volunteering, or communal recreation, constitutes a form of social capital that enhances one’s position within the social field. The persistence of employment among a segment of the older people reflects not only economic necessity but also the retention of symbolic capital associated with productivity and autonomy. Meanwhile, the predominance of housework among women may reflect gendered dispositions embedded in the habitus, shaped by long-standing cultural norms and expectations. Volunteering and collective recreational activities, though less frequent, represent opportunities for the accumulation of symbolic capital, especially in contexts where such engagement is socially valorized. Their medium ranking suggests potential underutilization, possibly due to structural constraints or limited institutional support. Bourdieu’s concept of field helps illuminate how older people navigate the social space of aging—where participation is both shaped by and shapes their access to resources, recognition, and social legitimacy.

In summary, the data not only reflects behavioral patterns but also reveals the interplay between structure and agency in later life. Encouraging diverse forms of social participation among the older people in Saudi Arabia may thus serve not only health and well-being goals but also contribute to the redistribution of social capital and the reconfiguration of aging-related habitus.

Table 7 reveals a relatively high overall mean score for social relationships among the older people (M = 2.5805), indicating a generally positive perception of social connectedness. Among the indicators assessed, “feelings about others” ranked highest (M = 2.68), followed closely by “communication with children” (M = 2.67) and “psychological normalcy in social contact” (M = 2.64). “Communication with friends” and “interaction with other relatives” followed with means of 2.61 and 2.60, respectively. Meanwhile, “relations with neighbors” and “relations with partners” were rated lower, at 2.53 and 2.33, respectively.

**Table 7:** Indicators of social relationships.

Items (*N* = 660)	Quality			Descriptive statistics		
	Poor	Acceptable	Good	Mean	Std. deviation	Rank
1. Communication with children	26	158	476	2.67	0.544	2
2. Communication with friends	36	188	436	2.61	0.590	4
3. Communication with other relatives	32	202	426	2.60	0.581	5
4. Relationships with neighbors	64	184	412	2.53	0.666	6
5. Relationship with partner	138	168	354	2.33	0.800	7
6. Normal psychological in social contact	16	204	440	2.64	0.528	3
7. Feelings about others	20	170	470	2.68	0.527	1
Average mean = 2.5805 (High)						

0.00–1.00 = Low; 1.01–2.00 = Moderate; 2.01–3.00 = High.

These findings underscore the layered nature of social relationships and their critical role in shaping health outcomes and quality of life, particularly in later stages of life. As Laura et al. (61) argue, social ties exert influence across the lifespan, with heightened significance in middle and older age. Grace and Samuel (43) further emphasize that the efficacy of social support—rather than mere connectedness—is a stronger predictor of well-being among older adults. This may explain the older people’s prioritization of relational quality over quantity. Bahramnezhad et al. (44) affirm that addressing the social needs of older individuals is a societal imperative, best approached through a nuanced understanding of their social networks and lived experiences.

From the perspective of Bourdieu’s theory of practice, the older people’s prioritization of emotionally significant relationships, particularly with children and close friends, can be interpreted as a strategic mobilization of social capital within the field of aging. These ties are not merely functional but carry symbolic weight, reinforcing the individual’s dignity, identity, and social positioning. The relatively lower scores for relationships with neighbors and partners may reflect diminished symbolic capital in those domains, possibly due to cultural norms, widowhood, or reduced mobility. Furthermore, the ranking of relational categories may mirror a generational habitus—a system of internalized dispositions shaped by long-standing cultural and familial expectations. In collectivist societies such as Saudi Arabia, this habitus often privileges familial bonds over peripheral social ties, shaping how older adults perceive and engage with their social environment. Applying spillover theory, the data suggests that strong emotional and familial relationships generate positive spillover effects into broader domains of psychological well-being. The high mean scores for interactions with children and feelings about others indicate that intimate relational domains contribute positively to the older people’s overall social satisfaction and mental health. Conversely, the lower engagement with neighbors and partners may reflect negative spillovers or constrained boundaries between private and public spheres. These findings imply that the quality of primary relationships can either buffer or exacerbate the effects of weaker ties in secondary social domains, influencing the overall experience of aging.

From a cognitive perspective, the older people’s relational preferences may be shaped by selective attention and schema-driven evaluations that prioritize emotional depth, predictability, and psychological security. As cognitive resources decline with age, older adults tend to optimize their social interactions by focusing on relationships that offer meaningful engagement and emotional support. This aligns with the Selective Optimization with Compensation (SOC) model, which posits that individuals adapt to aging by narrowing their social focus and compensating for losses through emotionally rewarding ties. The data reflects this cognitive prioritization, with higher scores for emotionally salient relationships and lower scores for more peripheral or less emotionally resonant connections.

Table 8 presents a relatively high mean score for the ego system among older people participants (M = 2.5280), suggesting a generally positive self-perception and psychological orientation. This dimension reflects the cumulative impact of social health indicators that contribute to the overall quality of life in later adulthood. Among the subcomponents, “self-health concern” ranked highest (M = 2.69), followed by “attitude towards life” (M = 2.63), “family financial security” (M = 2.44), and “interests and hobbies” (M = 2.35). These results indicate that older people prioritize health awareness and psychological outlook, with financial stability and personal engagement also playing meaningful roles.

**Figure 2:**
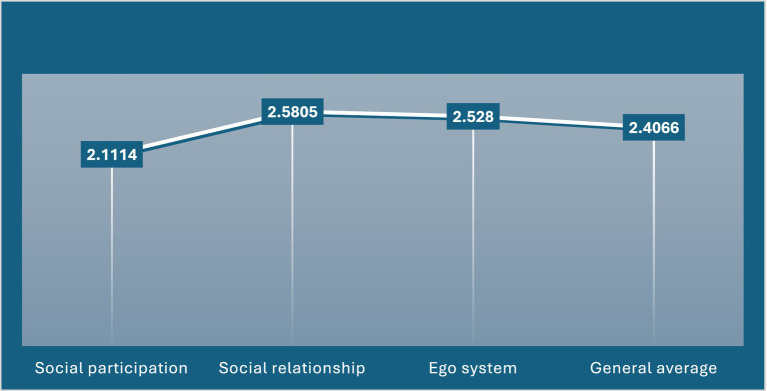
Social adjustment indicators.

Figure 2 presents a comparative overview of four key social adjustment indicators, highlighting meaningful variation in how older adults engage socially. The data show that social relationship scores are the strongest (2.5805), suggesting that older individuals maintain relatively solid interpersonal connections and supportive interactions. The ego system indicator follows closely (2.528), reflecting a generally positive sense of self perception and psychological stability. The general average (2.4066) indicates a moderate overall level of social adjustment across domains. In contrast, social participation records the lowest value (2.1114), pointing to more limited involvement in community or group activities. Taken together, the pattern suggests that while older adults demonstrate strong relational and psychological adjustment, their active participation in broader social settings may be comparatively constrained, highlighting an area where targeted interventions could enhance overall social well being.

**Table 8:** Indicators of ego system.

Items (*N* = 660)	Quality			Descriptive statistics		
	Poor	Acceptable	Good	Mean	Std. deviation	Rank
1. Self-health concern	70	66	524	2.69	0.654	1
2. Interests and hobbies	164	102	394	2.35	0.852	4
3. Family financial security	128	112	420	2.44	0.797	3
4. Attitude towards life	78	86	496	2.63	0.685	2
Average mean = 2.5280 (High)						

0.00–1.00 = Low; 1.01–2.00 = Moderate; 2.01–3.00 = High.

The findings align closely with Erik Erikson’s theory of psychosocial development, particularly the final stage of “ego integrity versus despair,” which characterizes late adulthood. According to Erikson, successful navigation of this stage results in a sense of mastery, self-worth, and psychological coherence—often expressed through self-esteem and spiritual integration. The prominence of “self-health concern” and “attitude towards life” in the data suggests that older people who maintain a proactive stance toward their well-being and cultivate a positive life outlook are more likely to experience ego integrity. Conversely, lower scores in areas such as hobbies may reflect diminished engagement or reduced opportunities for self-expression, which Erikson associates with feelings of inadequacy or despair. Prior research (45–47) supports the notion that individuals aged 80–90 tend to exhibit greater psychological and spiritual integration, reinforcing the idea that late-life development is not merely a decline but a potential culmination of identity and meaning. From a cognitive standpoint, the ego system indicators reflect how older people process, evaluate, and prioritize aspects of their identity and well-being. The prominence of “self-health concern” and “attitude towards life” suggests that older adults engage in self-referential cognition, where thoughts about one’s health and existential outlook become central to psychological functioning. This aligns with theories of cognitive appraisal, which posit that individuals interpret life events and internal states through subjective evaluations that shape emotional and behavioral responses. Moreover, the relatively lower scores for “interests and hobbies” may indicate a narrowing of attentional focus—a phenomenon consistent with selective attention and cognitive resource allocation in aging. As cognitive capacity becomes more constrained, older adults tend to invest in domains that offer emotional stability and existential coherence, reinforcing the adaptive nature of ego development in later life.

Social adjustment among older people is a critical determinant of well-being and life satisfaction in later adulthood. Empirical evidence suggests that both physical and social stressors significantly hinder the older people’s ability to adapt to their environment, thereby affecting their psychological resilience and social functioning. Al-Otaibi (48) highlighted the pivotal role of the family in facilitating successful social adaptation, noting its contribution to enhanced life satisfaction among older adults in the Kingdom of Saudi Arabia. Similarly, Adam (49) underscored the effectiveness of professional intervention programs—particularly the life model within generalist social work practicing promoting adaptive capacities among the older people. Furthermore, Abdul Hakim (50) emphasized that the formation of positive and meaningful relationships constitutes the most essential social service for fostering successful adjustment in old age.

### Differences in social health indicators promoting quality of life for the older people in the Kingdom of Saudi Arabia

The results in Tables 8–10 showed that there were statistically significant differences between all social health indicators promoting quality of life for the older people in the Kingdom of Saudi Arabia; between the main two dimensions (Social Support and Social Adjustment), and between sub-dimensions of social support (Emotional, Informational, and Instrumental), as well as between and sub-dimensions of social adjustment (Social Participation, Social Relation, and Ego System).

Reasons for the Differences According to the ANOVA Tables:

1) Differences in Social Support Indicators (Table 9):

A. The variations among emotional, informational, and instrumental support reflect the diverse psychological and social needs of older adults.
B. The higher valuation of emotional and informational support compared to instrumental support may be attributed to the fact that older people individuals place greater importance on forms of care that foster acceptance and social integration, while reliance on material support is less emphasized due to the strong family-based caregiving culture in Saudi Arabia.

2) Differences in Social Adjustment Indicators (Table 10):

A. The differences among social participation, social relationships, and ego systems highlight the varying patterns of social engagement among the older people.
B. Paid work and household tasks scored higher than volunteering and recreational activities, which may be explained by cultural and structural factors such as limited institutional opportunities for volunteering or leisure.
C. Family relationships (with children and relatives) ranked higher than relationships with neighbors or partners, reflecting the centrality of kinship ties in Saudi culture.

3) Differences between the Two Main Dimensions (Table 11):

A. The differences between social support and social adjustment suggest that quality of life among the older people is more strongly influenced by direct support (emotional and informational) than by adjustment processes, which require active participation and may be constrained by age, health, or gender-related factors.
B. This indicates that social support exerts a stronger impact on psychological satisfaction and social integration, while social adjustment is more dependent on physical capacity and available opportunities.

**Table 9:** Differences in social support indicators.

ANOVA						
(*N* = 660)		Sum of squares	df	Mean square	*F*	Sig.
Emotional support	Between groups	61.764	64	0.965	36.938	0.000
	Within groups	15.545	595	0.026		
	Total	77.309	660			
Informational support	Between groups	104.870	64	1.639	103.353	0.000
	Within groups	9.433	595	0.016		
	Total	114.303	660			
Instrumental support	Between groups	150.700	64	2.355	66.363	0.000
	Within groups	21.112	595	0.035		
	Total	171.812	660			

**Table 10:** Differences in social adjustment indicators.

ANOVA						
(*N* = 660)		Sum of squares	df	Mean square	*F*	Sig.
Social participation	Between groups	242.423	89	2.724	28.112	0.000
	Within groups	55.230	570	0.097		
	Total	297.653	660			
Social relation	Between groups	97.233	89	1.093	41.074	0.000
	Within groups	15.161	570	0.027		
	Total	112.394	660			
Ego system	Between groups	159.082	89	1.787	33.273	0.000
	Within groups	30.621	570	0.054		
	Total	189.703	660			

**Table 11:** Differences between the two dimensions social support and social adjustment.

ANOVA					
(*N* = 660)	Sum of squares	df	Mean square	*F*	Sig.
Between groups	77.257	89	0.868	21.706	0.000
Within groups	22.795	570	0.040		
Total	100.052	660			

The statistically significant differences revealed by the ANOVA tables reflect the influence of cultural, social, and economic factors in shaping patterns of support and adjustment among older adults in Saudi Arabia. Emotional and informational support emerge as the most critical contributors to quality of life, whereas social adjustment is more contingent upon structural opportunities and individual health conditions.

### Strengths and limitations

This study seeks to contribute to the understanding of social health indicators and quality of life among the older people in Saudi Arabia, within the broader framework of national efforts to enhance well-being as emphasized in Saudi Vision 2030. Its findings align with governmental initiatives in older people care, including the Quality-of-Life Program, expansion of health and social services, and promotion of community engagement for older adults. Thus, the study adds to Arab and international literature as a modest knowledge contribution that can support the alignment of policies and programs with sustainable development goals and Vision 2030’s commitment to dignity and well-being for the older people.

While this study contributes to understanding social health indicators and quality of life among the older people in Saudi Arabia, certain limitations should be acknowledged. Reliance on quantitative indicators using a specific instrument limited the exploration of subjective and qualitative experiences, and the gender imbalance in the sample (74.5% female vs. 25.5% male) may affect the generalizability of findings. These limitations highlight the need for future research that employs qualitative and longitudinal approaches and achieves greater gender balance, thereby strengthening the alignment of findings with Saudi Vision 2030 initiatives and programs aimed at enhancing quality of life and older people care.

## Conclusion

The findings of this study, which highlight the high quality of social health indicators among the older people in the Kingdom of Saudi Arabia, can be interpreted through multiple cultural, structural, and theoretical lenses:

1) Cultural Background of Older People Care: Although the study design did not include direct questions about Islamic teachings or religious beliefs, the interpretation of the findings related to the quality of social health indicators among the older people can benefit from being placed within a broader cultural context. Saudi society has historically been characterized by religious and social values that elevate the status of older adults and emphasize their respect and care. This cultural framework is not presented here as an empirically derived conclusion from the study data, but rather as a contextual lens that helps explain the continued significance of the older people and the reinforcement of intergenerational relationships in the Kingdom.
2) Family-Based Care as a Structural Norm: Despite a growing older people population—659,871 individuals aged 65 and above as of the 2022 census—formal institutional care remains limited, with only 12 social care homes nationwide. The fact that only 0.06% of the older people resides in these facilities, most of whom are single, illiterate, and without family support, affirms that elder care in Saudi Arabia is predominantly family-based. This reflects a deeply embedded social structure in which familial responsibility is both a legal and moral imperative. From Bourdieu’s perspective, the family operates as a primary field where social capital is accumulated and exchanged, ensuring the older people’s continued inclusion and support.
3) Demographic Shifts and Policy Imperatives: With global projections indicating that the proportion of individuals aged 65 and above will rise from 10% in 2022 to 16% by 2050 (51), Saudi Arabia is expected to experience parallel demographic transitions. This underscores the urgency of developing sustainable policies that monitor and enhance the social and health-related quality of life for the aging population.
4) Resilience of Social Value Amid Modernization: Despite the transformative effects of modernization on social relationships, the older people in Saudi society continue to hold significant social value. Studies have shown that Saudi youth, particularly those from extended families and rural areas, maintain positive attitudes toward older adults. This suggests the persistence of intergenerational solidarity and the transmission of cultural values that uphold the status of the older people. From a cognitive perspective, such attitudes may be shaped by early-life schemas and emotional bonds—especially with grandparents—that reinforce respect and empathy toward aging.
5) Evolving Family Structures and Enduring Obligations: Although the Saudi family has undergone structural and functional changes—such as the redefinition of parent–child dynamics and the rise of individual autonomy—care for the older people remains a core familial duty. This duty is not only legal and moral but also social, ensuring the preservation of dignity and fulfillment of rights for older adults. Empirical studies confirm that older people living within family settings report fewer psychological, material, and leisure-related problems. The high quality of life observed in such contexts, particularly in the cognitive and health dimensions, reflects the enduring role of the family as a site of emotional and symbolic investment.
6) Preparing for an Aging Future: Although Saudi Arabia is currently classified as a young society, projections suggest that older adults will constitute one-fifth of the population within the next three decades. This demographic shift necessitates proactive planning to address the emerging needs of this growing segment. From a cognitive standpoint, aging is accompanied by shifts in attention, memory, and emotional regulation. Therefore, policies must not only address physical and economic needs but also support cognitive well-being and social integration, ensuring that older people continue to experience meaning, autonomy, and belonging.

### Recommendations

The study demonstrates that older adults in Saudi Arabia enjoy relatively high social health indicators, supported by strong cultural values and family-based care structures. However, demographic transitions necessitate proactive measures to sustain and enhance this quality of life. Therefore, it is recommended to strengthen family-based support systems through financial incentives, caregiver training, and community awareness programs; expand healthcare services tailored to geriatric needs, including accessible primary care, mental health support, and preventive screening; and develop social assistance programs that address vulnerable groups—such as single, illiterate, or unsupported elders—by providing housing, social clubs, and outreach services. Moreover, promoting intergenerational solidarity via educational curricula and youth engagement initiatives is essential to reinforce respect and empathy toward older adults. Finally, planning for demographic shifts by integrating aging-related priorities into national health strategies will ensure sustainable resource allocation for long-term care. In conclusion, safeguarding the dignity and well-being of older adults requires coordinated action between lawmakers, healthcare practitioners, and society at large. By embedding cultural values within modern policy frameworks, Saudi Arabia can ensure that its aging population continues to experience autonomy, belonging, and meaningful social participation.

## Data Availability

The datasets presented in this study can be found in online repositories. The names of the repository/repositories and accession number(s) can be found at: https://doi.org/10.5281/zenodo.10893857; https://doi.org/10.5281/zenodo.10893866.
