# Correction: Social health indicators promoting quality of life for the older people in the Kingdom of Saudi Arabia

**DOI:** 10.3389/fpubh.2026.1839959

**Published:** 2026-04-07

**Authors:** Hanaa Faize A. Moubarak, Basheer Ali Allouash, Abeer Niazi Wajeed Fathallah, Asyraf Afthanorhan

**Affiliations:** 1 Department of Social Sciences, College of Arts, University of Ha'il, Ha'il, Saudi Arabia; 2 Humanities Research Center, University of Ha'il, Ha'il, Saudi Arabia; 3 Faculty of Business and Management, Universiti Sultan Zainal Abidin (UniSZA), Kuala Terengganu, Malaysia

**Keywords:** older people, quality of life, social adjustment, social health, social support

Author Hanaa Faize A. Moubarak was erroneously assigned to affiliation “Higher Institute of Social Work, Alexandria, Egypt”. This affiliation has now been removed for author Hanaa Faize A. Moubarak.

Author Abeer Niazi Wajeed Fathallah was erroneously assigned to affiliation “Capital University, Helwan, Egypt”. This affiliation has now been removed for author Abeer Niazi Wajeed Fathallah.

The original version of this article has been updated.

